# Presence of a Mitovirus Is Associated with Alteration of the Mitochondrial Proteome, as Revealed by Protein–Protein Interaction (PPI) and Co-Expression Network Models in *Chenopodium quinoa* Plants

**DOI:** 10.3390/biology11010095

**Published:** 2022-01-08

**Authors:** Dario Di Silvestre, Giulia Passignani, Rossana Rossi, Marina Ciuffo, Massimo Turina, Gianpiero Vigani, Pier Luigi Mauri

**Affiliations:** 1 Laboratory of Proteomics and Metabolomics, Institute for Biomedical Technologies (ITB), Department of Biomedical Sciences, National Research Council (CNR), 20054 Milan, Italy; giulia.passignani@itb.cnr.it (G.P.); rossana.rossi@itb.cnr.it (R.R.); pierluigi.mauri@itb.cnr.it (P.L.M.); 2 Institute for Sustainable Plant Protection, Department of Bio-Food Sciences, National Research Council (CNR), 10135 Turin, Italy; marina.ciuffo@ipsp.cnr.it (M.C.); massimo.turina@ipsp.cnr.it (M.T.); 3 Plant Physiology Unit, Department of Life Sciences and Systems Biology, University of Turin, 10135 Turin, Italy

**Keywords:** proteomics, mitochondrion, PPI, co-expression, network, virus, quinoa, systems biology

## Abstract

**Simple Summary:**

Plants often harbor persistent plant virus infection transmitted only vertically through seeds, resulting in no obvious symptoms (cryptic infections). Several studies have shown that such cryptic infections provide resilience against abiotic (and biotic) stress. We have recently discovered a new group of cryptic plant viruses infecting mitochondria (plant mitovirus). Mitochondria are cellular organelles displaying a pivotal role in protecting cells from the stress of nature . Here, we look at the proteomic alterations caused by the mitovirus cryptic infection of *Chenopodium quinoa* by Systems Biology approaches allowing one to evaluate data at holistic level. Quinoa is a domesticated plant species with many exciting features of abiotic stress resistance, and it is distinguished by its exceptional nutritional characteristics, such as the content and quality of proteins, minerals, lipids, and tocopherols. These features determined the growing interest for the quinoa crop by the scientific community and international organizations since they provide opportunities to produce high-value grains in arid, high-salt and high-UV agroecological environments. We discovered that quinoa lines hosting mitovirus activate some metabolic processes that might help them face drought. These findings present a new perspective for breeding crop plants through the augmented genome provided by accessory cryptic viruses to be investigated in the future.

**Abstract:**

Plant mitoviruses belong to *Mitoviridae* family and consist of positive single-stranded RNA genomes replicating exclusively in host mitochondria. We previously reported the biological characterization of a replicating plant mitovirus, designated Chenopodium quinoa mitovirus 1 (CqMV1), in some *Chenopodium quinoa* accessions. In this study, we analyzed the mitochondrial proteome from leaves of quinoa, infected and not infected by CqMV1. Furthermore, by protein–protein interaction and co-expression network models, we provided a system perspective of how CqMV1 affects mitochondrial functionality. We found that CqMV1 is associated with changes in mitochondrial protein expression in a mild but well-defined way. In quinoa-infected plants, we observed up-regulation of functional modules involved in amino acid catabolism, mitochondrial respiratory chain, proteolysis, folding/stress response and redox homeostasis. In this context, some proteins, including BCE2 (lipoamide acyltransferase component of branched-chain alpha-keto acid dehydrogenase complex), DELTA-OAT (ornithine aminotransferase) and GR-RBP2 (glycine-rich RNA-binding protein 2) were interesting because all up-regulated and network hubs in infected plants; together with other hubs, including CAT (catalase) and APX3 (L-ascorbate peroxidase 3), they play a role in stress response and redox homeostasis. These proteins could be related to the higher tolerance degree to drought we observed in CqMV1-infected plants. Although a specific causative link could not be established by our experimental approach at this stage, the results suggest a new mechanistic hypothesis that demands further in-depth functional studies.

## 1. Introduction

Mitoviruses belong to *Mitoviridae* family inside the Lenarviricota phylum and consist of positive single-stranded RNA (ssRNA) genomes. Most mitoviruses replicate exclusively in host mitochondria. It is suggested they are derived from an ancestral mitochondrial phage by losing the capsid protein [1]. In fact, they are naked viruses presenting only one open reading frame (ORF) that encodes one protein, the RNA-dependent RNA polymerase (RdRp) [2]. By investigating complete plant and fungal genomes, the presence of nonretroviral endogenous RNA viral elements has been revealed in almost all of the eukaryotic nuclear genomes [3,4,5]. In particular, Bruenn et al. demonstrated the presence of mitoviral sequences in many plant nuclear and mitochondrial genomes [5], which was the first indirect evidence that plants, at some point, could also become infected by mitoviruses; more recently, further indirect evidence of replicating plant mitoviruses has been provided by mining the transcriptome of a number of plant species [6]. In this scenario, we have previously reported the complete genome sequence and the biological characterization of a replicating plant mitovirus, designated Chenopodium quinoa mitovirus 1 (CqMV1), in some *Chenopodium quinoa* accessions [7]. Mitoviruses were first discovered and characterized in fungi, but recently, in addition to their occurrence in some plant mitochondria, evidence of mitoviruses infecting insects was also provided [8].

Based on the knowledge that mitoviruses typically infect, replicate and persist in the mitochondria [9], including our own specific fractionation studies on CqMV1 [7], in this study we evaluated the quinoa mitochondrial proteome modulation, due to CqMV1 infection. For this goal, we combined proteomic and systems biology approaches based on network analysis [10]. To date, few works have focused on quinoa proteome by shotgun approaches [11,12,13], while none have relied on protein–protein interaction (PPI) and/or protein co-expression (Co-Exp) network analysis. This is mainly due to the lack of a quinoa PPI network model and accurate functional annotations [14]. However, the number of studies focusing on PPIs prediction in plants is constantly increasing [15,16,17]. PPI network reconstruction often relies on orthologs in model plants, such as *Arabidopsis thaliana* [14,18]. Although this approach presents some limitations, ranging from false positives to poor coverage from associalogs, it represents a valuable strategy for a rapid PPI network inference in plant organisms [19]. Alternatively, and complementarily to PPI, Co-Exp models, reconstructed from experimental proteomic data, represent a way to model large-scale proteomic datasets of non-model plants as networks [18,20].

In addition, to characterize the mitochondrial proteome from leaves of quinoa infected (+CqMV1) and not infected (−CqMV1) by CqMV1, we reconstructed quinoa PPI and Co-Exp network models by homology with Arabidopsis. These models were processed to identify hubs and differentially correlated proteins, as well as functional modules whose expression was affected by mitovirus infection [10,18]. Thus, we here intend to provide a molecular and system perspective of how CqMV1 affects mitochondrial functionality, shedding light on proteins with a key role in the response to viral infection within mitochondria.

## 2. Materials and Methods

### 2.1. Plant Materials

Plants used in this study corresponded to two virus-infected lines (cv Regalona and accession IPSP; +CqMV1 lines) and two virus-free ones (BO78 and BO25; −CqMV1 lines). Source of these lines has been previously described [7]. Plants were grown in a commercial soil substrate in 9 cm diameter pots in insect-proof greenhouses with a maximum temperature set at 28 °C and minimum set at 20 °C, with natural light supplemented in winter with fluorescent lamps. The 10 youngest and most fully developed leaves were harvested from each plant 4 weeks after transplant, when plants had an approximate height of 40 cm. Drought stress was established on plants (4 weeks after transplant) by stopping water supply for 4 days, and relative water content (RWC) was determined [21].

### 2.2. Mitochondrial Enrichment Protocol

Mitochondrion-enriched fractions were obtained by a modified protocol already used for *Cucumis sativus* [22]. Ten grams of quinoa leaves was homogenized at a ratio of 1:10 in chilled extraction buffer (0.45 M sucrose, 15 mM morpholine propanesulfonic acid (MOPS), 1.5 mM EGTA, pH 7.4, with KOH) with 0.6% polyvinylpyrrolidone (PVP), 0.2% bovine serum albumin (BSA), 10 mM dithiothreitol and 0.2 mM phenylmethylsulfonyl fluoride (PMSF). After filtration on Miracloth (Calbiochem), the homogenate was centrifuged at 2000× *g* for 5 min to separate chloroplasts and cell debris. The supernatant was again centrifuged at 13,000× *g* for 30 min to obtain the mitochondrial fraction in the pellet. The crude mitochondrial pellet was resuspended in 1 mL of washing buffer (WB; 0.6 M sucrose, 20 mM MOPS, 2 mM EGTA, pH 7.2, with KOH) with 0.2 mM PMSF, layered on a Percoll gradient (18%, 23% and 40% in WB), and centrifuged at 12,000× *g* in an SW21 rotor (Beckmann) for 45 min. The mitochondrial fraction, between the 23% and 40% interface, was collected with a Pasteur pipette, diluted in 30 mL of SRM buffer, and centrifuged at 2000× *g* for 5 min to remove all the Percoll. This step was repeated two times. We resuspended the pellet in about 0.1 mL WB and checked the quality of purification by observation with a fluorescence microscope [7]. Finally, the mitochondrial-enriched fractions were stored at −80 °C until use for protein extraction and LC-MS/MS analysis.

### 2.3. Sample Preparation for LC-MS

The enriched mitochondrial fractions were centrifuged at 12,000× *g* for 20 min and treated with RapiGest^TM^ SF reagent (Waters Co, Milford, MA, USA) at the final concentration of 0.25% (*w*/*v*). The resulting suspensions were incubated under stirring at 100 °C for 20 min. Subsequently, the samples were cooled at room temperature and centrifuged 10 min at 2200× *g*. The protein concentration was assayed using the SPN^TM^ Protein Assay kit (G-Biosciences, St. Louis, MO, USA), and 50 ± 0.5 μg protein from each sample was digested with Sequencing-Grade Modified Trypsin (Promega Inc., Madison, WI, USA) using a 1:50 (*w*/*w*) enzyme/substrate ratio at 37 °C overnight. The next morning, an additional aliquot of trypsin (1:100 *w*/*w*) was added at an enzyme/substrate ratio of 1:100 (*w*/*w*) and the digestion continued for 4 h. Enzymatic digestion was chemically stopped with the addition of 0.5% trifluoroacetic acid (TFA) (Sigma-Aldrich Inc., St. Louis, MO, USA), and a subsequent incubation at 37 °C for 45 min completed the RapiGest^TM^ SF acid hydrolysis. Water immiscible degradation products were removed by centrifugation at 13,000 rpm for 10 min. Finally, the digested mixtures were desalted using PierceTM C-18 spin columns (Thermo Fisher Scientific, Pierce Biotechnology, Rockford, IL, USA), concentrated in a SpeedVac (Savant Instruments Farmingdale, NY, USA) at 60 °C and resuspended in 0.1% formic acid (Sigma-Aldrich Inc., St. Louis, MO, USA) in water (LC-MS Ultra CHROMASOLV^TM^, Honeywell Riedel-de HaenTM, Muskegon, MI, USA) at a concentration of 0.2 μg/μL.

### 2.4. Nano LC-MS/MS Analysis

Enriched mitochondrial fractions from leaves of quinoa lines, not infected (BO25, BO78; *n* = 6 per line) and infected (IPSP, Regalona (REG); *n* = 6 per line), for a total of 24 LC-MS/MS runs, were analyzed by LC-MS/MS; specifically, *n* = 3 biological replicates × *n* = 2 technical replicates per line were considered. Trypsin-digested mixtures were analyzed by means of a platform consisting of the Eksigent nanoLC-Ultra 2D System (Eksigent, AB SCIEX, Dublin, CA, USA), configured in trap-elute mode, coupled with a high-resolution mass spectrometer. Samples were first loaded on the nanoLC trap (200 μm × 500 μm ChromXP C18, 3 μm, 120 Å) and washed with the loading pump running in isocratic mode with 0.1% formic acid in water for 10 min at a flow rate of 3 μL/min. The automatic switching of nanoLC ten-port valve then eluted the trapped mixture on a nano-reversed phase column (75 μm × 15 cm 3C18-CL, 3 μm, 120 Å), through a 140 min gradient of eluent B (eluent A, 0.1% formic acid in water; eluent B, 0.1% formic acid in acetonitrile), at a flow rate of 300 nL/min. Specifically, gradient was: from 5–10% B in 3 min, 10–50% B in 104 min, 50–95% B in 20 min and holding at 95% B for 13 min. Mass spectra were acquired using a LTQ-Orbitrap XL-ETD mass spectrometer (Thermo Fisher Scientific, San José, CA, USA), equipped with a nano-spray ionization source (Thermo Fisher). The spray capillary voltage was set at 1.7 kV, and the ion transfer capillary temperature was held at 220 °C. Full mass spectra were recorded in positive ion mode over a 400–1600 m/z range, resolution setting of 30000 FWHM and scan rate of 2 spectra per second, followed by five low-resolution MS/MS events sequentially generated in a data-dependent manner on the top five most intense ions selected from the full MS spectrum (at 35% collision energy), using dynamic exclusion of 0.5 min for MS/MS analysis. Mass spectrometer scan functions and high-performance liquid chromatography solvent gradients were controlled by the Xcalibur data system version 1.4 (Thermo Fisher Scientific, Monza, Italy).

### 2.5. Processing of Raw Mass Spectra

All raw files produced by LC-MS/MS were processed by the SEQUEST HT algorithm contained in Proteome Discoverer 2.5 software (Thermo Fisher Scientific, San José, CA, USA). Experimental MS/MS spectra were compared with the theoretical mass spectra obtained by in silico digestion of a *Chenopodium quinoa* protein database containing 63173 sequences (www.ncbi.nlm.nih.gov, accessed on 1 May 2021) plus *Chenopodium quinoa mitovirus 1* RNA-dependent RNA polymerase (RdRp) (www.uniprot.org, accessed on 1 May 2021). The following searching criteria were set: trypsin enzyme, maximum number of missed cleavages per peptide was set to 3, mass tolerances of ±50 ppm for precursor ions and ±0.8 Da for fragment ions. Percolator node was used with a target-decoy strategy to give a final false discovery rate (FDR) ≤0.01 based on *q*-values, considering maximum deltaCN of 0.05. Only peptides with minimum peptide length of 5 amino acids, confidence at “Medium” level and rank 1 were considered. Protein grouping and strict parsimony principle were applied.

### 2.6. Protein Profiles Preprocessing, Statistical Evaluations and Quantitative Analysis

Due to the high redundancy rate of sequences in *Chenopodium quinoa* fasta file, identified protein sequences were compared by Clustal Omega Tool [23]. Pairwise alignment score cutoff (≤0.1) was considered for redundancy evaluation; an *in house* R script, based on splitstackshape and dplyr libraries, was used to collapse redundant sequences. Selected distinct proteins were processed by STRING [24] and by UNIPROT BLAST to retrieve *Arabidopsis thaliana* homologous proteins/genes. Subcellular localization of the identified quinoa proteins was predicted by Plant-mSubP tool [25,26]. It predicts 11 single locations (cell membrane, cell wall, plastid, cytoplasm, endoplasmic reticulum, extracellular, golgi apparatus, mitochondrion, nucleus, peroxisome and vacuole) and takes into consideration also three significant multi location proteins (cytoplasm-nucleus, mitochondrion-plastid and cytoplasm-golgi apparatus). Specifically, we used the PseAACNCCDipep module of Plant-mSubP because it considers the hybrid feature of the pseudo amino acid composition, and in comparison to other Plant-mSubP modules PseAACNCCDipep showed the best overall accuracy. In addition, to increase the selection of putative mitochondrial proteins we have taken into consideration also the UNIPROT gene ontology (GO) cellular component (CC) of *Arabidopsis thaliana* homologous proteins.

Spectral counts (SpCs) of the identified proteins were normalized using a total signal normalization method [27] and compared using a label-free quantification approach based on SpCs [28]. Data matrix dimensionality (24 samples × 2186 distinct proteins) was reduced by linear discriminant analysis (LDA); a pairwise comparison (−CqMV1 vs. +CqMV1) was performed and proteins with *p*-value ≤ 0.05 were retained. Fold change (by DAve index) [29] of proteins selected by LDA was estimated by comparing average −CqMV1 and +CqMV1 spectral counts (avSpCs); positive DAve values indicate proteins up-regulated in −CqMV1 plants, whereas negative DAve values indicate proteins up-regulated in +CqMV1. Differentially expressed proteins (DEPs) selected by LDA were processed by hierarchical clustering by applying the *Ward*’s method and the *Euclidean* distance metric. In addition, SpCs of DEPs were processed by *Spearman*’s rank correlation and principal component analysis (PCA). All processing were performed by JMP15.2 SAS software.

### 2.7. Chenopodium quinoa Protein–Protein Interaction (PPI) and Co-Expression (Co-Exp) Network Model Reconstruction

A *Chenopodium quinoa* PPI network model was reconstructed by homology with *Arabidopsis thaliana*, as previously reported [18]. Specifically, mitochondrial predicted or annotated *Chenopodium quinoa* protein sequences (*n* = 515) were submitted to STRING Cytoscape’s App [30] aiming to homology with Arabidopsis thaliana proteins. Each Chenopodium quinoa protein sequence was associated with Arabidopsis thaliana protein, showing the best identity value. For reconstructing the *Chenopodium quinoa* PPI network model, all *Arabidopsis thaliana* homologous proteins (*n* = 506) were considered regardless their homology, and their physical and/or functional interactions were filtered by considering only those “experiments” or/and “databases” annotated, with a STRING Score ≥0.15 and ≥0.35, respectively.

Reconstructed networks were analyzed at topological level by Analyzer App integrated in Cytoscape v.3.8.2 [31]. Using the same approach, a second PPI network was reconstructed starting from mitochondrial DEPs; DEPs were grouped in functional modules by the support of the GO enrichment tool inserted in STRING Cytoscape’s App [30].

Protein Co-Exp networks were reconstructed by processing −CqMV1 (*n* = 12) and +CqMV1 (*n* = 12) quinoa mitochondrial protein profiles. To reduce the number of missing values, only proteins (*n* = 165) with identification frequency (IF)≥ 70% were retained and processed; of note, 119 out of 165 proteins were identified in all analyzed samples ((IF) ≥ 100%). −CqMV1 and +CqMV1 protein data matrices were processed by using *Spearman*’s rank correlation coefficient; *p* ≤ 0.01, corresponding to a *Spearman*’s rank correlation score ≥ $\left|0.7\right|$, was set as threshold. All processing was performed using the statistical software JMP15.2 SAS, while the reconstructed Co-Exp networks were visualized and analyzed by Cytoscape platform and its Apps [31].

Both PPI and Co-Exp networks were topologically analyzed by Centiscape Cytoscape’s App [32]. As for PPI networks, Betweenness and Bridging centralities were calculated, and nodes with above-average values were considered PPI hubs [18]. A set of differentially correlated proteins (DCPs) were selected from Co-Exp network based on degree centrality, and nodes with an above-average degree were considered Co-Exp hubs. In addition, −CqMV1 (or +CqMV1) hubs with a degree higher than twice average, and a degree in +CqMV1 (or −CqMV1) lower than half average, were considered −CqMV1 (or +CqMV1) best Co-Exp hubs, respectively.

Statistical significance of all topological results was tested by considering randomized network models; they were reconstructed and analyzed by an *in house* R script based on VertexSort (to build random models), igraph (to compute centralities), and ggplot2 (to plot results) libraries; results were visualized in the form of Violin plots.

### 2.8. Real-Time PCR

For CqMV1 detection, leaves of quinoa lines tested were placed in extraction bags (Bioreba, Reinach, Switzerland) and diluted 1:20 (*w*/*v*) with carbonate buffer pH 9.6 (94) added with 2% PVP40, 0.2% BSA, 1% sodium metabisulfite and 0.05% tween 20. Raw extract WAS diluted 1:10 in sterile water and boiled 10 min at 95 °C. qRT-PCR screening was performed using a CFX96™ Real-Time PCR Detection System (Biorad). PCR mix was prepared with iTaq™ Universal Probes Supermix (Biorad) adding 3 U of reverse transcriptase from High-Capacity RNA-to-cDNA Kit (Thermo Fisher Scientific, San José, CA, USA) for each sample. Reactions were performed in 10 μL of total volume adding 1 μL of boiled extract to 9 μL of PCR mix. The qRT-PCR protocol had a 30 min step at 37 °C to perform the reverse transcription of the viral genome, then was followed by 1 min at 94 °C and 40 steps of denaturation at 95 °C for 10 s, annealing, and extension at 60 °C for 30 s.

## 3. Results

**Figure 1:**
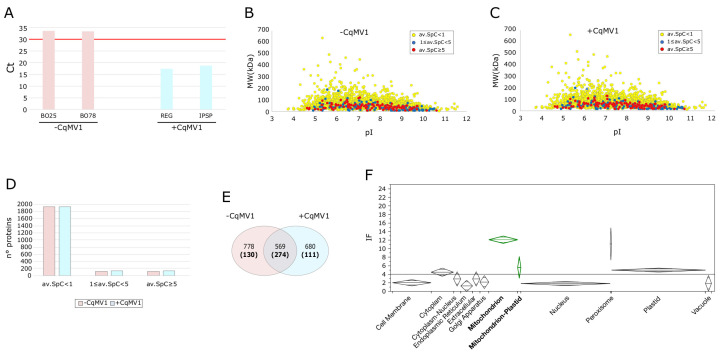
Mitochondrial proteome from leaves of *Chenopodium quinoa*, infected and not infected by CqMV1. (**A**) qRT-PCR assay resulting in a Ct (threshold cycle) of 17.3 and 18.6 for IPSP and REG lines, respectively, whereas for BO25 and BO78 the Ct were beyond the specificity threshold (≥30). (**B**,**C**) Virtual 2D map (pI vs. MW) of proteins identified in –CqMV1 and +CqMV1 lines, respectively; the colour code indicates average SpC (av.SpC) per protein. (**D**) N° proteins identified by av.SpC ≤ 1, 1 ≥ av.SpC ≤ 5 and av.SpC ≥ 5. (**E**) Venn diagram of proteins identified in −CqMV1 and +CqMV1; proteins annotated or predicted as mitochondrial are shown in bold and in brackets. (**F**) Cellular components (CC) of proteins identified in mitochondria enriched fractions from leaves of quinoa; average identification frequency per sub-cellular localization is shown (IF; out of 24 LC-MS runs).

In this study, we investigated how viral infection by CqMV1 affects quinoa mitochondrial protein abundance and estimated the effect on processes and functions. For this purpose, we analyzed the mitochondrial proteome from leaves of quinoa lines, not infected (BO25, BO78) and infected (IPSP, REG), after confirming presence/absence of CqMV1 by a specific qRT-PCR assay [7] (Figure 1A). In addition, to identify differentially expressed proteins (DEPs) between not infected and infected phenotypes, data were evaluated at functional level by systems biology approaches based on graph theory, and in particular based on protein–protein interaction (PPI) and Co-Exp network models. For this goal, due to missing functional annotations concerning quinoa proteins, we relied on homology with Arabidopsis and its annotations.

### 3.1. Mitochondrial Proteome from Leaves of Chenopodium quinoa, −CqMV1 and +CqMV1

The combination of 24 LC-MS/MS runs (BO25, BO78, IPSP, REG; *n* = 6 per line) of proteins extracted from enriched quinoa mitochondrial fractions allowed for the identification of 2186 total distinct proteins (Figure 1B–E). About 10% of proteins (*n* = 244) were characterized by a total average SpC ≥ 1 and high rate of identification (Identification Frequency, IF) (Table S1). To improve our knowledge about the subcellular localization of the identified proteins, we performed their prediction by processing the quinoa protein sequences (Table S2). We found that predicted (or/and annotated) mitochondrial proteins showed the highest rate of identification (average IF = 12), while it was much lower for proteins classified in other cellular components. These findings fit with the enrichment of mitochondria and suggest a randomized identification of proteins that are not mitochondrial. Thus, for further functional evaluations, we exclusively focused our attention on the mitochondrial proteins here characterized. Globally, 515 proteins were classified as mitochondrial or mitochondrial-plastidial (Figure 1E,F, Table S3); 274 were found in both conditions, while 128 and 106 were specifically identified in −CqMV1 and +CqMV1 lines, respectively.

### 3.2. Mitochondrial Proteins Differentially Expressed in −CqMV1 vs. +CqMV1

**Figure 2:**
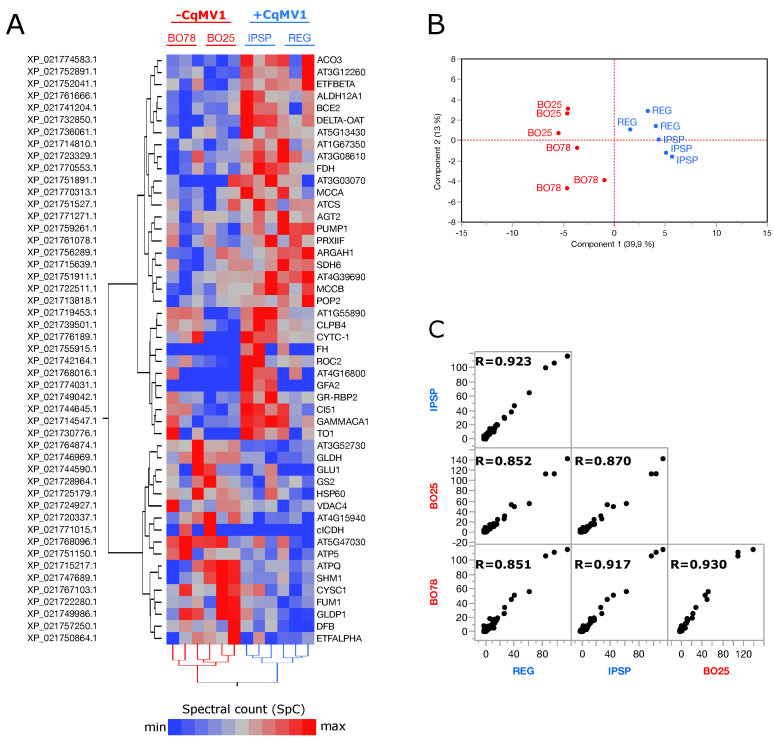
Label-free quantification. (**A**) Hierarchical clustering of differentially expressed mitochondrial proteins (DEPs) selected by comparing −CqMV1 and +CqMV1 quinoa (*p*≤ 0.05), *Ward*’s method and *Euclidean distance* were used (JMP15.2 SAS software); for each DEP, protein RefSeqs NCBI accession and *Arabidopsis thaliana* gene name (average homology 73%, min homology 39%, max homology 91%) are shown. (**B**) Principal component analysis and (**C**) *Spearman*’s correlation by processing the spectral count (SpC) of DEPs.

Following the comparison between −CqMV1 and +CqMV1 protein profiles, 49 mitochondrial proteins resulted in differentially expressed proteins (DEPs) (Figure 2A, Table S4). As shown by the heat map, some proteins (*n* = 16) were also differentially expressed between the two cultivars within not-infected (BO25 vs. BO78, n DEPs = 11) and infected (IPSP vs. REG, n DEPs = 11) quinoa lines (Table S4). In particular, ATP synthase subunit d (ATPQ) and serine hydroxymethyltransferase (SHM1) were specifically up-regulated in BO25, while Chaperone protein dnaJ (GFA2) was specifically identified in IPSP. All other DEPs followed a trend correlated to presence or absence of virus infection; in fact, all biological replicates were grouped based on their phenotype (−CqMV1 and +CqMV1) (Figure 2A,B), and correlation scores were higher between cultivars sharing the presence or absence of virus infection (Figure 2C).

**Figure 3:**
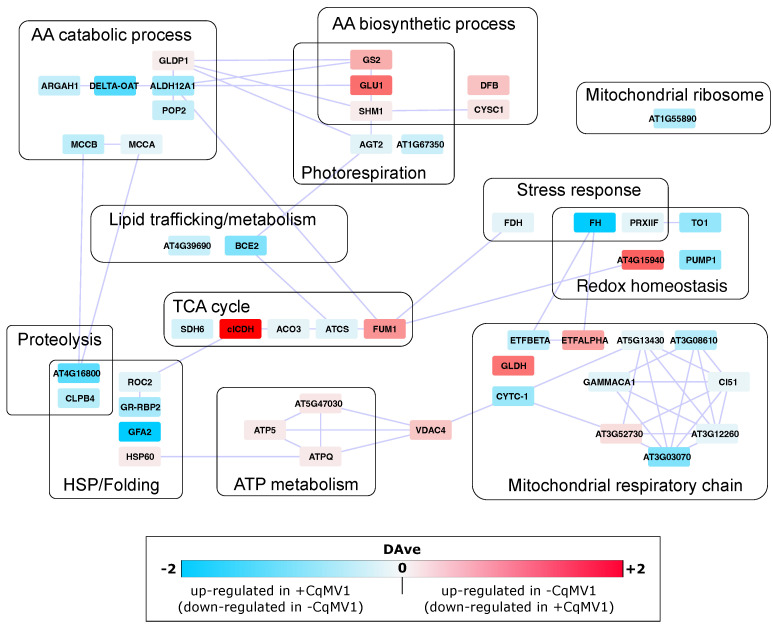
Differentially expressed PPI functional modules between −CqMV1 and +CqMV1 quinoa. Functional modules were enriched by STRING Cystoscap’s App. Positive DAve values (shade of red) indicate proteins up-regulated in −CqMV1 (and down-regulated in +CqMV1), while negative DAve values (shade of blue) indicate proteins up-regulated in +CqMV1 (and down-regulated in −CqMV1).

DEPs were grouped in 12 PPI functional modules. Even if differential protein expression did not show large differences in terms of fold change (DAve index), proteins/nodes belonging to the same module overall showed a well-defined expression trend (Figure 3). Globally, most modules were related to metabolism (amino acids, lipids, ATP, and TCA cycle). Those most consistent in terms of node number were mitochondrial respiratory chain and amino acid metabolism. Other modules influenced by virus infection were involved in proteolysis, HSP/Folding, redox homeostasis and stress response; all of them were up-regulated in +CqMV1 (and down-regulated in −CqMV1), along with mitochondrial respiratory chain and amino acid catabolic process. On the contrary, amino acid biosynthetic process and ATP metabolism were down-regulated in +CqMV1 (and up-regulated in −CqMV1). Of note, an opposite trend emerged concerning amino acid catabolic and biosynthetic processes, as well as between ATP metabolism and mitochondrial respiratory chain.

Concerning DEPs, most of them were identified in both −CqMV1 and +CqMV1 phenotypes. However, FH (frataxin) and GFA2 (chaperone protein dnaJ GFA2) were exclusively found in +CqMV1, while cICDH (cytosolic NADP+-dependent isocitrate dehydrogenase) was the only DEP exclusively found in −CqMV1.

### 3.3. Network Analysis

**Figure 4:**
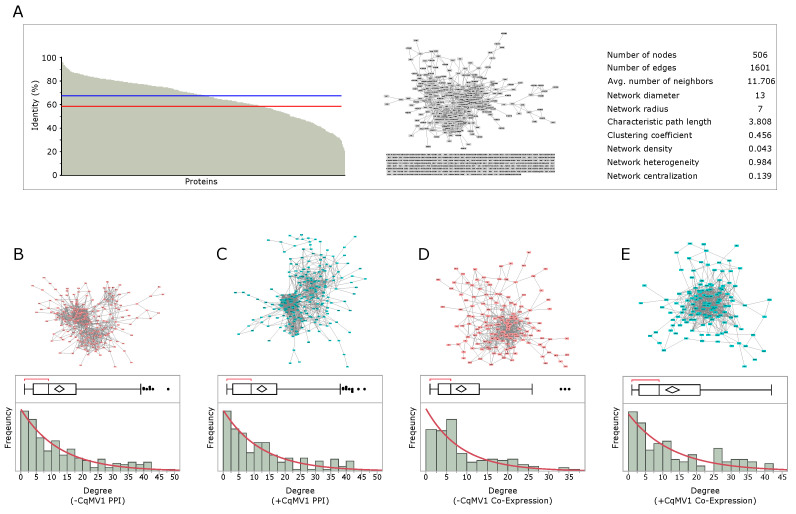
*Chenopodium quinoa* protein–protein interaction (PPI) and co-expression network reconstruction. (**A**) Sequence homology between mitochondrial predicted/annotated *Chenopodium quinoa* proteins and *Arabidopsis thaliana* (*n* = 506); red line indicates the average sequence homology (58%), while blue line indicates median (67.5%). Using exclusively predicted or/and annotated mitochondrial proteins, a PPI model of 506 nodes and 1601 edges was reconstructed by STRING (only experiments’ (score ≥ 0.15) or databases’ (score ≥ 0.35) annotated interactions were considered). Network topological parameters, by Cytoscape 3.8.2 NetworkAnalyzer, are shown. PPI network and degree distribution in (**B**) −CqMV1 and (**C**) +CqMV1 quinoa. Co-expression networks and degree distribution in (**D**) −CqMV1 and (**E**) +CqMV1 quinoa.

Starting from the identified and classified mitochondrial proteins, we reconstructed PPI and Co-Exp quinoa network models by homology with Arabidopsis, as previously described for *Cucumis sativus* [18]. Of 515 mitochondrial quinoa proteins, 506 were associated to an Arabidopsis protein, while 9 sequences did not return any match. Globally, quinoa sequences showed an average sequence homology with Arabidopsis equal to 58%, and 90% of proteins showed identity values ranging from 20.7 to 96.8, with a median equal to 67.5% (Figure 4A, Table S3). By exploiting Arabidopsis PPIs, a quinoa PPI network model of 506 nodes and 1601 edges was built. In parallel, mitochondrial proteins with higher rate of identification (IF ≥ 70%, *n* = 165) were processed by *Spearman*’s correlation, and −CqMV1 and +CqMV1 quinoa Co-Exp network models were reconstructed.

The major connected PPI network modules of −CqMV1 (217 nodes and 1350 edges) and +CqMV1 (218 nodes and 1356 edges) lines, as well as the major connected −CqMV1 (148 nodes and 637 edges) and +CqMV1 (127 nodes and 779 edges) co-expression modules, showed a degree distribution typical of scale-free networks (Table 1, Figure 4B–E). In comparison to −CqMV1, a preliminary topological analysis evidenced in +CqMV1 co-expression model a higher network density and a lower network diameter, indicating a more compact network that can be interpreted as the overall easiness of the proteins to communicate and/or influence their reciprocal function. In contrast, no significant differences were observed between the −CqMV1 and +CqM1 PPI networks; in fact, a high value of network diameter in −CqMV1 PPI model can be misleading in terms of evaluation of graph compactness because it is possible that two nodes are very distant, thus giving a high graph diameter, but several other nodes are not. Therefore, a graph could have high diameter and still being rather compact or have very compact regions (Table 1) [33].

#### *Chenopodium quinoa* PPI and Co-Expression Network Hubs

**Figure 5:**
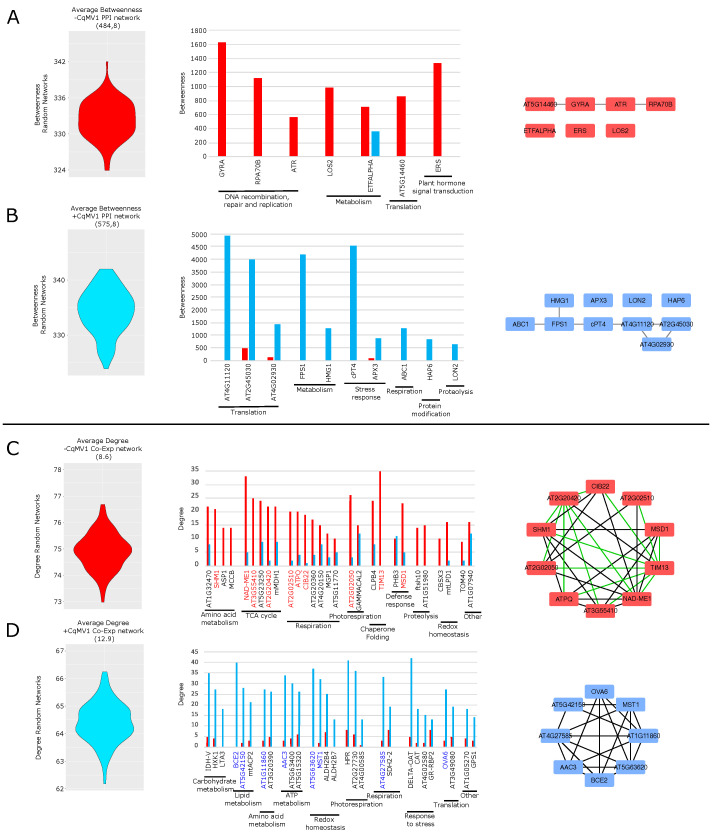
Protein–protein interaction (PPI) and co-expression (Co-Exp) network hubs. PPI hubs in −CqMV1 (**A**,**B**) +CqMV1 network models. Violin plots show average betweenness in −CqMV1 (in red) and +CqMV1 (in blue) PPI random network models. Selected hubs are functionally grouped and sorted by descending betweenness. Hubs in −CqMV1 (**C**,**D**) +CqMV1 co-expression network models. Violin plots show average degree in −CqMV1 (in red) and +CqMV1 (in blue) Co-Exp random network models. Selected hubs are functionally grouped and sorted by descending degree. Red and blue highlighted gene names indicate best hubs (selected by high stringent criteria) in −CqMV1 and +CqMV1 Co-Exp networks, respectively. Red and blue highlighed nodes represent Co-Exp hubs defined “best”, while green network edges indicate negative correlations.

PPI networks of −CqMV1 and +CqMV1 lines, and the corresponding Random models, were analyzed and compared at topological level by considering betweenness and bridging centralities. Nodes with both centralities above average in −CqMV1 PPI (and both centralities below average in +CqMV1 PPI) were defined −CqMV1 PPI hubs (Figure 5A, Table S5). Similarly, nodes with both centralities above average in +CqMV1 PPI (and both centralities below average in −CqMV1 PPI) were defined +CqMV1 PPI hubs (Figure 5B, Table S5). Most −CqMV1 PPI hubs were involved in DNA recombination, repair and replication, such as GYRA (DNA gyrase subunit A), RPA70B (replication protein A 70 kDa DNA-binding subunit B) and ATR (serine/threonine-protein kinase ATR), while from +CqMV1 PPI hubs stress response (cPT4, cis-prenyltransferase 4; APX3, L-ascorbate peroxidase 3) and translation mainly emerged, such as AT4G11120 (elongation factor Ts), AT4G02930 (elongation factor Tu) and AT2G45030 (Elongation factor G-2) (Figure 5A,B). In addition, in both phenotypes some hubs were metabolism-related.

Using the same strategy adopted for PPI models but considering degree centrality, we selected a group of proteins differentially correlated (DCPs or Co-Exp hubs) between −CqMV1 and +CqMV1 Co-Exp models. Concerning −CqMV1, most Co-Exp hubs were involved in mitochondrial respiration, TCA cycle and amino acid metabolism (Figure 5C, Table S6). On the contrary, carbohydrate metabolism, lipid metabolism, ATP metabolism, stress response and redox homeostasis were the functional categories characterizing most +CqMV1 Co-Exp hubs (Figure 5D, Table S6).

Although no hub was in both PPI and Co-Exp models, we found that some of the hubs were also differentially expressed proteins (Table 2). Specifically, BCE2 (lipoamide acyltransferase component of branched-chain alpha-keto acid dehydrogenase complex), DELTA-OAT (ornithine aminotransferase) and GR-RBP2 (glycine-rich RNA-binding protein 2) were up-regulated in +CqMV1 and, simultaneously, +CqMV1 Co-Exp hubs; of note, both DELTA-OAT and GR-RBP2 proteins are involved in response to stress. Similarly, ATPQ (ATP synthase subunit d), SHM1 (serine hydroxymethyltransferase 1) and ETFALPHA (electron transfer flavoprotein subunit alpha) were up-regulated in −CqMV1; ATPQ and SHM1 were also −CqMV1 Co-Exp hubs, while ETFALPHA was hub in −CqMV1 PPI. On the contrary, MCCB (methylcrotonoyl-CoA carboxylase beta chain) and CLPB4 (chaperone protein ClpB4) were up-regulated in +CqMV1 and Co-Exp hubs in −CqMV1.

### 3.4. Abiotic Stress Differentially Affected Infected and Not Infected Line

**Figure 6:**
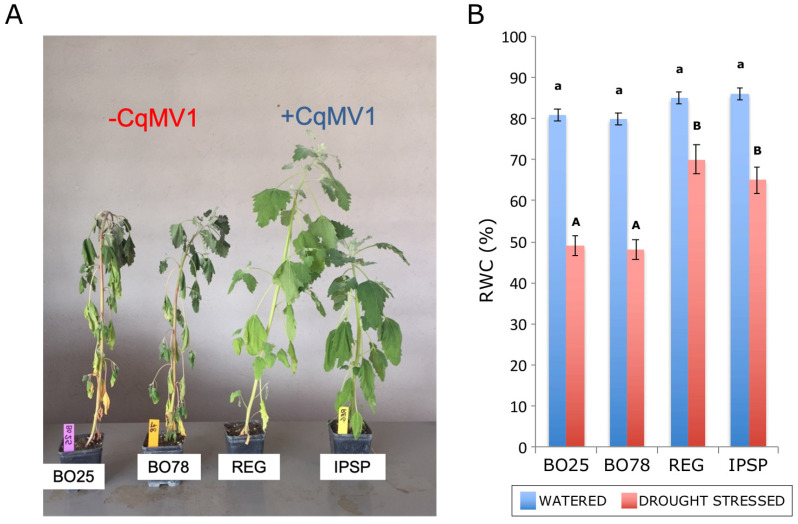
Infected and not infected lines affected by drought stress. (**A**) Effect of drought stress on −CqMV1 (BO25, BO78) and +CqMV1 (REG and IPSP) lines (scale bar: pot width 10 cm). (**B**) Average values of relative water content (RWC) of leaves under watered (blue bars) and drought (red bars) conditions. Different letters indicate statistically significant difference (*p*≤ 0.05) within the watered (lowercase letters) and drought stress (capital letters) samples of 5 independent biological replicates (*n* = 5); Anova and Tukey’s test were used.

Since proteomic and network analysis revealed up-regulation of stress-responsive proteins in CqMV1 infected lines, we aimed to test the impact of an exemplary abiotic stress (drought) on the quinoa lines considered. Water stress was determined by avoiding water supplementation for 4 days. After this period, differential wilting symptoms were observed in quinoa lines: −CqMV1 plants (BO25 and BO78 lines) displayed a more severe wilting status of leaves compared with +CqMV1 plants (REG and IPSP) (Figure 6A). Accordingly, the relative water content (RCW) was significantly higher in CqMV1-infected plants compared with not-infected lines (Figure 6B), while the RCW determined on both upper and lower leaves under well-watered condition did not reveal any differences among the quinoa lines considered (data not shown).

## 4. Discussion

Following our recent findings about existence of plant mitochondrial viruses as true virus-encoded RdRp-dependent replicating RNA elements [7], we provided here molecular evidence, at proteomic system level, that *Chenopodium quinoa Mitovirus 1* is associated with changes in mitochondrial protein expression in *Chenopodium quinoa*-infected plants in a mild but well defined way. Given the wealth of reports on quinoa molecular characterization, it is somewhat surprising that its proteome has received only limited attention. In fact, this study is among the few manuscripts that provide features of quinoa proteome by large-scale proteomic approaches [11,12,13] and the first focused on its mitochondrial proteome, as well as the first quinoa proteome investigation relying on systems biology and graph theory.

In addition to characterizing mitochondrial proteins of quinoa leaves, the proteomic analysis had the aim of measuring a differential physiological reaction between CqMV1 infected and not infected plants. Globally, by their quantitative comparison, marked differences were not detected. In fact, although 49 DEPs represent about 10% of all identified and predicted mitochondrial proteins, most fold change variations were low. This could fit with the idea that mitovirus seems to cause no specific harm to its host [7], and its presence was unnoticed phenotypically even though quinoa has been a common test plant among virology labs worldwide, earning the name “cryptic” for its infection features. Although *Chenopodium quinoa* mitochondrial virus is not subjected to the typical dicer/argonaute-dependent antiviral silencing response [7,34], mitochondrial virus replication in plants is likely limited by the generation of specific sRNA produced through other molecular pathways. In this context, we previously found that 16-nt mitoviral sRNA results from a nonviral specific RNA degradation process [7], while other authors hypothesized a sRNA production driven by pentatricopeptide repeat proteins (PPR proteins), providing a testable model of antiviral defense based on intra-mitochondrial sRNA generation [35,36].

Even if we did not observe a considerable differential expression between −CqMV1 and +CqMV1 quinoa lines, some PPI functional modules showed a well-defined expression trend. Noteworthily, it was opposite for amino acid catabolic and biosynthetic processes, as well as between ATP metabolism and mitochondrial respiratory chain, suggesting non-random regulation; similarly, PPI network hubs suggest in infected plants an activation of translation at the expense of DNA recombination, repair and replication and thus a coordination of these mechanisms by virus infection. Moreover, the increase of amino acid catabolism in infected plants correlated with up-regulation of proteins involved in proteolysis and HSP/folding. Notably, +CqMV1 lines displayed an increase of some proteins belonging to the branched chain amino acid (BCAA) catabolism (BCE2, MCCB), as well as arginine catabolism (DELTA-OAT). It has been demonstrated that catabolism of BCAAs (aa belonging to the aspartate family) functions as an alternative electron donor in the respiratory chain under stress condition [37]. Of note, BCAAs also play an important role in plant drought tolerance as an alternative source of respiratory substrates; under energy-limited conditions, such amino acid family can be degraded in mitochondria providing electrons to the respiratory chain, and in precursors to the TCA [38]. In the same way, in sesame genotypes, drought tolerance traits have been linked to the ability of plants to properly modulate redox homeostasis as well as amino acid metabolism (e.g., induction of BCAA catabolism) [39].

As for ornithine aminotransferase (DELTA-OAT), whose expression is increased by CqMV1 infection, it has been reported to enhance tolerance to multiple abiotic stress [40,41]. It is a pyridoxal phosphate (PLP)-dependent enzyme involved in the conversion of ornithine (Orn) into pyrroline-5-carboxylate (P5C), using α-ketoglutarate (AKG) and glutamate (Glu) as co-substrates [42]. Since P5C is a precursor of proline, it has been suggested that DELTA-OAT belongs to the alternative route of proline biosynthesis in plants via arginine catabolism. DELTA-OAT is also important for recycling Orn in plants. Ornithine is a non-protein amino acid playing a central role in the polyamine (PA) amino acid biosynthetic pathway; indeed, it has been suggested that ornithine might be involved in the monitoring and/or signalling pathway for the biosynthesis of metabolites such as proline, putrescine, γ-aminobutyric acid (GABA), and perhaps also arginine [43].

Infected plants also showed up-regulation of a module involved in stress response and redox homeostasis. This is in agreement and reinforces our previous speculation that the presence of CqMV1 in the mitochondria alters the oxidative stress cellular signalling [7]. It was further suggested by network analysis with the identification of a number of +CqMV1 PPI (cPT5, APX3) and Co-Exp (DELTA-OAT, CAT, AT4G02580 and GR-RBP2) hubs involved in stress response and redox homeostasis. The enzymatic defense activity of catalase (CAT) against viruses has been reported in several studies [44,45,46,47], while L-ascorbate peroxidase 3 (APX3) has been recently associated with protection from oxidative damage in cross-protected plants [48]. The efficient defence against oxidative stress at cellular and subcellular levels may be related to the high tolerance of plants to environmental stresses [49]. In this context, mitochondria play a key role defending themselves and the cell from an excess of ROS. In fact, mitochondria represent a major source of ROS production and consequent oxidative damage in the plant cell, as indicated by proteomic studies [50,51]. To do this, the continuous consumption/regeneration of small antioxidant molecules (such as ascorbate, glutathione and NADPH) and the modulation of ROS detoxyfying enzymes are required. In addition, among differentially expressed hubs, GR-RBP2 was interest because, in *Arabidopsis thaliana* under cold stress, it was observed to exert its function by modulating the expression and activity of various classes of genes, including catalase and peroxidase [40].

## 5. Conclusions

Although the combination of high-throughput proteomics and network analysis represents a powerful tool to decipher the proteome modulation induced by plant–microorganism interaction, its application to non-model organisms, such as quinoa, displays some limitations. As already reported in the introduction section, many plant species, including quinoa, suffer from the lack of well-annotated protein sequences, functional annotations and network models that would allow facing these system studies in a more complete and reliable way. Nevertheless, besides representing the first large-scale study on the mitochondrial proteome from leaves of quinoa, this work provided new insights on how CqMV1 affects mitochondrial functionality by identifying proteins (differentially expressed and network hubs) modulated by viral infection and potentially involved in the plant response. In this scenario, we revealed that CqMV1-infected plants (REG and IPSP) show up-regulation of proteins involved in amino acid catabolism, folding/stress response and redox homeostasis. The modulation of such processes occurs in plants growing under several stress conditions, including drought. Notably, the up-regulation of these processes in +CqMV1 lines mirrors the higher water retention and higher tolerance degree to drought observed in CqMV1 infected plants, although a direct link between the presence of CqMV1, the modulation of specific proteins and the ability of REG and IPSP to cope with drought stress could not be established by our experimental approach at this stage. However, it represents a new mechanistic hypothesis deserving in-depth investigation in the near future, through a greater number of samples and multi-omics data, to improve the reconstruction of the network models, and with new experiments, to validate the most relevant proteins predicted by the network analysis (in particular, differentially expressed hub proteins that represent central point of regulation of metabolic pathways and cellular processes characterizing specific phenotypes).

## Figures and Tables

**Table 1:** Topological analysis of −CqMV1 (in red) and +CqMV1 (in blue) PPI and Co-Exp network models.

TOP. PARAMS	PPI NETWORK		Co-Exp NETWORK	
	−CqMV1	+CqMV1	−CqMV1	+CqMV1
Connected components	1	1	1	1
Numbers of nodes	217	218	151	130
Numbers of edges	1350	1356	651	837
Avg. number of neighbors	12.442	12.440	8.623	12.877
Network diameter	9	13	12	8
Network radius	5	7	6	5
Characteristic path length	3.245	3.654	3.708	2.963
Clustering coefficient	0.494	0.457	0.354	0.415
Network density	0.058	0.057	0.057	0.100
Network heterogeneity	0.938	0.933	0.866	0.899
Network centralization	0.166	0.156	0.166	0.156

**Table 2:** Proteins both differentially expressed and hubs in −CqMV1 (in red) and +CqMV1 (in blue).

Differential Expression		PPI HUBS		Co-Exp HUBS	
DEPS	UP-reg in	−CqMV1	+CqMV1	−CqMV1	+CqMV1
BCE2	+CqMV1				X
DELTA-OAT	+CqMV1				X
MCCB	+CqMV1			X	
CLPB4	+CqMV1			X	
GR-RBP2	+CqMV1				X
ATPQ	−CqMV1			X	
SHM1	−CqMV1			X	
ETFALPHA	−CqMV1	X			

## Data Availability

The raw files corresponding the proteomic datasets generated and analyzed for this study can be found in the MassIVE repository (https://massive.ucsd.edu, at the following link ftp://massive.ucsd.edu/MSV000088052/.

## Supplementary Materials

The following are available online at https://www.mdpi.com/article/10.3390/biology1010000/s1, Table S1: Protein identified in mitochondria enriched fractions from leaves of Chenopodium quinoa, −CqMV1 and +CqMV1, Table S2: Subcellular localization prediction of proteins identified in mitochondrial enriched fractions from leaves of Chenopodium quinoa, −CqMV1 and +CqMV1, Table S3: Mitochondrial predicted or annotated proteins identified in mitochondria enriched fractions from leaves of Chenopodium quinoa, −CqMV1 and +CqMV1, Table S4: Differentially expressed proteins, selected by LDA (*p* < 0.05), by comparing mitochondrial proteomes from leaves of Chenopodium quinoa, −CqMV1 and +CqMV1 (*n* = 12 per condition), Table S5: −CqMV1 (in red) and +CqMV1 (in blue) PPI hubs, Table S6: −CqMV1 (in red) and +CqMV1 (in blue) Co-Expression hubs.
